# Foamy Virus Budding and Release

**DOI:** 10.3390/v5041075

**Published:** 2013-04-10

**Authors:** Sylvia Hütter, Irena Zurnic, Dirk Lindemann

**Affiliations:** 1 Institute of Virology, Medical Faculty "Carl Gustav Carus", Technische Universität Dresden, Fetscherstr. 74, Dresden 01307, Germany; E-Mails: Sylvia.huetter@mailbox.tu-dresden.de (S.H); irena.zurnic@tu-dresden.de (I.Z.); 2 DFG-Center for Regenerative Therapies Dresden (CRTD)—Cluster of Excellence, Technische Universität Dresden, Fetscherstr. 105, Dresden 01307, Germany

**Keywords:** foamy virus, budding, virus egress, capsid-glycoprotein interaction, pseudotyping, subviral particles

## Abstract

Like all other viruses, a successful egress of functional particles from infected cells is a prerequisite for foamy virus (FV) spread within the host. The budding process of FVs involves steps, which are shared by other retroviruses, such as interaction of the capsid protein with components of cellular vacuolar protein sorting (Vps) machinery via late domains identified in some FV capsid proteins. Additionally, there are features of the FV budding strategy quite unique to the spumaretroviruses. This includes secretion of non-infectious subviral particles and a strict dependence on capsid-glycoprotein interaction for release of infectious virions from the cells. Virus-like particle release is not possible since FV capsid proteins lack a membrane-targeting signal. It is noteworthy that in experimental systems, the important capsid-glycoprotein interaction could be bypassed by fusing heterologous membrane-targeting signals to the capsid protein, thus enabling glycoprotein-independent egress. Aside from that, other systems have been developed to enable envelopment of FV capsids by heterologous Env proteins. In this review article, we will summarize the current knowledge on FV budding, the viral components and their domains involved as well as alternative and artificial ways to promote budding of FV particle structures, a feature important for alteration of target tissue tropism of FV-based gene transfer systems.

## 1. Introduction

The step crucial for virus spread within the infected host is the egress of virus particles from the initial and any successively infected cell. Though there are variations of this process as virus spread both by release and subsequent infection of other cells or by direct transfer between neighboring cells are observed. During evolution, numerous virus genera have devised a variety of ways to ensure proper particle egress from the host cell, thus enabling infection of naïve cells and production of new virus progeny. 

The release of enveloped viruses, including retroviruses, from the cell surface, is a highly coordinated process, usually aided by a number of cellular factors [1]. Budding of viruses can occur simultaneously with virus assembly or start only after fully assembled, but immature, viral cores have formed in the cytoplasm. The subcellular location of virus budding also varies among virus genera and for specific virus species sometimes also in dependence of the target tissue type infected [2]. It can either take place at the plasma membrane or at an intracellular compartment. In the latter case, however, it is not yet fully clarified how the virus-containing vesicles exit the cell, the most often proposed mechanism being exocytosis.

**Figure 1:**
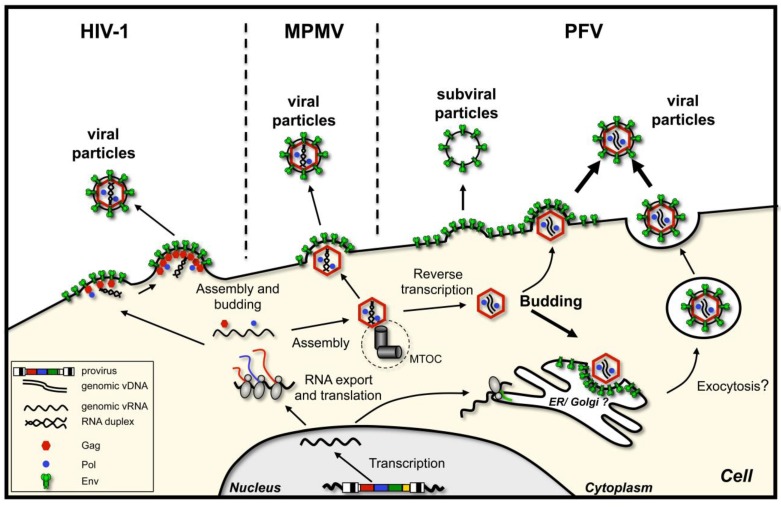
Retroviral capsid assembly and budding strategies. Schematic illustration of the capsid assembly and budding strategies of C-type (human immunodeficiency virus type 1, HIV-1), B/D-type (Mason-Pfizer monkey virus, MPMV), and foamy viruses (prototype foamy virus, PFV).

In general, retroviruses follow two budding pathways that are also represented in Figure 1:

1. Particle assembly occurs at the plasma membrane, in regions enriched in envelope glycoprotein. Oligomerization of capsid protein leads to formation of the particle, its growth and bending of the plasma membrane, after which the assembled virion pinches off. This is characteristic of C-type retroviruses, such as human immunodeficiency virus type 1 (HIV-1) [3] or murine leukemia virus (MuLV) [4]. As mentioned, here capsid assembly and virion budding occur simultaneously. However, importantly, neither the presence of the glycoprotein nor its interaction with the capsid is a prerequisite for virus release, although they might enhance particle release.
2. Immature capsids with encapsidated RNA genome assemble first in the cytoplasm. Then they travel to the budding site, in some cases assisted by the viral glycoprotein. The budding site can be either plasma membrane or an intracellular compartment (such as endoplasmic reticulum (ER) or Golgi), where the envelope protein is localized. This mechanism characterizes B/D-type retroviruses such as Mason-Pfizer monkey virus (MPMV) and mouse mammary tumor virus (MMTV) [5,6,7].

Interestingly, outside the retrovirus family, budding of Hepatitis B virus (HBV), a member of the reverse transcribing *Hepadnaviridae* family, shares some similarities with the B/D-type retroviruses, in that HBV cores assemble in the cytoplasm prior to being enveloped and released from the cell [8,9]. A major difference of HBV to orthoretroviruses is that the nucleocapsids contain viral DNA reverse transcribed from packaged viral pre-genomic RNA, which is a prerequisite for subsequent HBV core envelopment at the pre-Golgi compartment. The HBV virions bud through this cellular compartment and are finally released from the cell by exocytosis. Unlike most retroviruses (e.g., HIV-1, MuLV), HBV virions depend on the presence of the envelope proteins for successful budding from the cell.

## 2. Foamy Virus Budding, an Overview

Some years ago, retroviruses were reclassified into two separate subfamilies with spuma- or foamy viruses (FVs) as the only genus of the *Spumaretrovirinae* subfamily [10]. The realization that the FV replication strategy represents a link between *Retro-* and *Hepadnaviridae* is among the reasons leading to this reclassification and make FVs to interesting research subjects [11]. The FV budding strategy certainly strengthens this notion, as it combines steps that occur during budding of some retroviruses, with unique characteristics common to HBV. In this review, which summarizes the current knowledge on FV budding, most aspects are described for Prototype FV (PFV), originally known as human FV (HFV), the best studied FV species. However, if information is available for other FV species or there are known mechanistic differences between individual FV species, this will be addressed accordingly. Important features of the budding strategy of FVs in comparison to hepadnaviruses and orthoretroviruses are summarized in Table 1. 

**Table 1:** Features of HIV-1, RSV, PFV and HBV budding strategies.

Virus species	Retroviroviridae			Hepadnaviridae
	Human Immunodeficiency Virus Type 1 (HIV-1)	Rous Sarcoma Virus (RSV)	Prototype Foamy Virus (PFV)	Hepatitis B virus (HBV)
**Glycoprotein organization**	Polyprotein precursor (gp160): Surface glycoprotein (gp120^SU^) Transmembrane glycoprotein (gp41^TM^)	Polyprotein precursor (gp95): Surface glycoprotein (gp85^SU^) Transmembrane glycoprotein (gp37^TM^)	Polyprotein precursor (gp130): Surface glycoprotein (gp80 ^SU^) Transmembrane glycoprotein (gp48 ^TM^) Leader peptide (gp18^LP^)	Hepatitis B surface antigen (HBsAg): Small (S) protein (226aa) Middle (M) protein (226aa + preS2 domain 55aa) Large (L) protein (226aa + preS2 domain 55aa + preS1 domain 108aa or 119aa)
**Capsid organization**	Gag precursor (Pr55^Gag^): Matrix (p17^MA^); Capsid (p24^CA^); Spacer p2 Nucleocapsid (p7^NC^); Spacer p1; p6 domain	Gag-PR precursor (Pr76^Gag-PR^): Matrix (p17^MA^); p2a, p2b; pp10; Capsid (p27^CA^); Spacer; Nucleocapsid (p12^NC^); Protease (p15^PR^)	Gag precursor (p71^Gag^): p68^Gag^; p3^Gag^	Hepatitis B core protein (p22^HBc^): 183-185aa
**Capsid: membrane targeting domain**	Present in MA subunit	Present in MA subunit	No	Not known
**Budding type**	Type C morphogenesis: Assembly takes place at the plasma membrane; particle release from plasma membrane or plasma membrane-derived membranes	Type C morphogenesis: Assembly takes placeat the plasma membrane; particle release from plasma membrane or plasma membrane-derived membranes	Type B/D morphogenesis: Capsid preassembly at the MTOC in the cytoplasm; budding from intracellular membranes (ER/Golgi) and plasma membrane	Nucleocapsid-formation in cytosol; Budding from intracellular membranes (ER)
**ESCRT-dependent budding process **	Yes ESCRT I, III AIP1/Alix Vps4A/B	Yes ESCRT II, III AIP1/Alix Vps4A/B	Yes ESCRT I, III Vps4A/B	Yes ESCRT II, III Vps4A/B
**L domain**	Gag (p6): PTAP; YPXL	Gag (p2b): PPPY; LYPSL	Gag (p71, p68): PSAP	Core: PPAY; K96?
**ESCRT interaction partner**	Tsg101; AIP1/Alix;	AIP1/Alix; (Nedd4)	Tsg101	(Nedd4); (γ2-adaptin)
**Virus like particles**	Yes	Yes	No	No, but release of naked capsids
**Subviral particles**	No	No	Yes, low amounts	Yes, high amounts
**Budding requires**	Capsid (Gag) protein only	Capsid (Gag) protein only	Capsid (Gag) and Envelope protein (Env) necessary	Capsid (Core) and Envelope protein (L and S) necessary vDNA synthesis
**Place of interaction (Capsid-Envelope)**	plasma membrane	plasma membrane	trans-Golgi network	ER
**Pseudotyping**	yes	yes	yes, but only with a artificial heterodimerizer system	no

## 3. Glycoprotein-Dependent Particle Release

Unlike orthoretroviruses, but analogous to hepadnaviruses, a hallmark of FV egress and transmission to new host cells is the strict requirement of a very specific interplay between capsids and the cognate glycoprotein [12,13,14]. Association of FV capsids with or budding across membranes in the absence of Env coexpression is not observed [12,13,14,15]. Apparently, FV Gag proteins lack membrane-targeting domains (MTDs) that are inherent to orthoretroviral capsid proteins and enable VLP release. Not alone is Env co-expression important to direct Gag to cellular membranes, but also the presence of Gag is necessary for efficient transport of Env to the cell surface [16]. Furthermore, heterologous viral surface proteins fail to substitute the essential FV Env function in particle morphogenesis. These notions support the idea of a very specific and potential direct interaction of Gag and Env protein essential to the FV budding process [12,13,14]. Nevertheless, what are the underlying molecular mechanisms and details of this exceptional retroviral budding strategy? Some of the unique features of the FV egress strategy are based on the unusual biosynthesis and special characteristics of the FV structural proteins. 

### 3.1. FV Capsid- and Glycoprotein Biosynthesis

**Figure 2:**
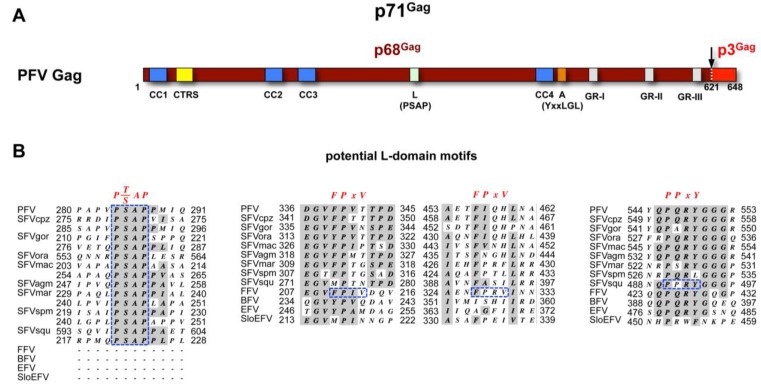
Foamy virus Gag. (**A**) Schematic organization of PFV Gag protein. The primary cleavage site within the p71^Gag^ precursor resulting in processing into p68^Gag^ and p3^Gag^ cleavage products is indicated by an arrow. CC1-4: putative coiled-coil motifs; CTRS: cytoplasmic targeting and retention signal; L: late assembly domain (PSAP); A: assembly domain (LYxxLGL); GR-I-III: glycine-arginine rich boxes I to III. Numbers indicate amino acid position in PFV Gag; (**B**) Sequence alignment of putative L-domain motifs of different FV species. Consensus L-domain motifs are shown in red on top. FV sequences matching the L-domain motif consensus are marked in dashed, blue boxes. Sequences used for alignment were retrieved from GenBank. PFV: prototype foamy virus, human foamy virus Y07725.1; SFVcpz: chimpanzee simian foamy virus, NC001364.1; SFVgor: gorilla simian foamy virus, HM245790.1; SFVora: orangutan simian foamy virus, AJ544579.1; SFVmac: macaques simian foamy virus, NC010819.1; SFVagm: African green monkey simian foamy virus, NC010820.1; SFVmar: common marmoset simian foamy virus, GU356395.1; SFVspm: spider monkey foamy virus, EU010385.1; SFVsqu: squirrel monkey foamy virus, GU356394.1; FFV: feline foamy virus, Y08851.1; BFV: bovine foamy virus, NC001831.1; EFV: equine foamy virus, NC_002201.1; SloEFV: sloth endogenous foamy virus, [23]. Complete Gag sequences were aligned using MacVector software and Gonnet matrix and sequence surrounding putative FPxV or PPxY L-domain motifs extracted. For PSAP L-domain motifs the core consensus motif together with four N- and C-terminal flanking amino acids extracted and manually aligned. Sequence identities are shaded in grey. Numbers indicate amino acid position in the respective full-length protein.

**FV capsid protein biosynthesis**. Like most of the viral structural proteins, FV Gag is expressed on free ribosomes in the cytoplasm. The subdomain structure and maturation of FV Gag proteins is quite different to other retroviral capsid proteins (see Müllers and Lee *et al*. in this issue for further details) (Figure 2A). In contrast to orthoretroviruses, only one primary protease cleavage site in the FV Gag precursor is utilized by the viral protease during assembly [17]. For PFV the 71 kDa Gag precursor is processed into a large p68^Gag^ subunit and a small p3^Gag^ protein. Both the Gag precursor and the large cleavage product are found in released FV particles at ratios of 1:1 to 1:4 [18,19,20,21]. Due to its small size the p3^Gag^ evaded so far detection by immunoblotting or immunoprecipitation techniques [22]. Therefore, it remains unclear whether p3^Gag^ constitutes a stable product of FV Gag precursor processing and may be a structural component of infectious viral particles. 

**Figure 3:**
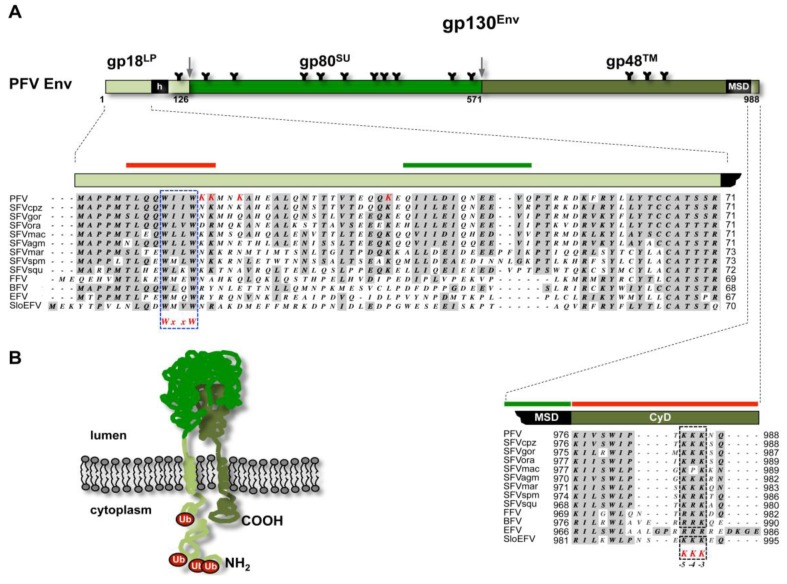
Foamy virus Env. (**A**) Schematic organization of PFV Env protein. The furin cleavage sites within the gp130^Env^ precursor that are used for generation of the mature gp18^LP^, gp80^SU^, and gp48^TM^ subunits are indicated by grey arrows. The individual subunits are shown as boxes in different shades of green. Hydrophobic sequences spanning the membrane in LP (h) and TM (MSD) subunit are indicated. Enlarged are the N-terminal cytoplasmic domain (CyD) of the LP subunit and the C-terminal CyD of the TM subunit with sequence alignment of different FV species. Domains reported to be essential for SVP release are marked by solid green bars and domains inhibiting SVP release are marked by solid red bars on top of the enlargements. Complete Env sequences of different FV species were aligned using MacVector software and Gonnet matrix. Sequence identities are shaded in grey. For the TM CyD sequences additional manual alignments were introduced to better illustrate the location of the ER retrieval signal (KKK), marked by dashed, blue boxes. The evolutionary conserved WxxW motif in the CyD of the LP subunit, important for the interaction with the capsid, is marked with a dashed, blue box. The lysine residues in PFV Env LP that were experimentally confirmed to be ubiquitinated are highlighted in red. Sequences used for alignment were retrieved from GenBank. PFV: prototype foamy virus, human foamy virus Y07725.1; SFVcpz: chimpanzee simian foamy virus, NC001364.1; SFVgor: gorilla simian foamy virus, HM245790.1; SFVora: orangutan simian foamy virus, AJ544579.1; SFVmac: macaques simian foamy virus, NC010819.1; SFVagm: African green monkey simian foamy virus, NC010820.1; SFVmar: common marmoset simian foamy virus, GU356395.1; SFVspm: spider monkey foamy virus, EU010385.1; SFVsqu: squirrel monkey foamy virus, GU356394.1; FFV: feline foamy virus, Y08851.1; BFV: bovine foamy virus, NC001831.1; EFV: equine foamy virus, NC_002201.1; SloEFV: sloth endogenous foamy virus, [23]. (**B**) Membrane topology of the PFV Env precursor gp130^Env^. N-terminus (NH2) and C-terminus (COOH) are indicated. Attached ubiquitin molecules (Ub) are indicated as red balls.

**FV glycoprotein biosynthesis**. The biosynthesis of the FV Env protein is unique in comparison to the other retroviral glycoproteins. The full-length FV Env precursor protein is translated at ribosomes of the endoplasmatic reticulum (ER) membrane and is inserted into membrane with N- and C-termini located in cytoplasm (Figure 3) [15,24,25]. FV glycoprotein precursor processing by cellular proteases displays analogies and differences to orthoretroviruses. Like in orthoretroviruses the central surface (SU) and C-terminal transmembrane (TM) subunits of FV are derived from the glycoprotein precursor by furin or furin like protease-mediated processing, probably occurring in late compartments of the Golgi (Figure 3A) [26,27]. All known FV Env proteins harbor consensus motifs of optimal furin cleavage sites (RX[K/R]R) between both subunits. SU/TM processing does not significantly influence particle egress but is essential for infectivity of released FV virions [16,28,29]. Similar to HIV Env, but different to other orthoretroviruses, FV Env proteins are not cotranslationally processed by the signal peptidase [15,24,30]. However, whereas HIV-1 Env is posttranslationally cleaved by signal peptidase, additional processing by furin or furin-like proteases was discovered for PFV Env or FFV proteins [26,27,30]. All known FV species contain one or multiple minimal (RxxR) or optimal furin cleavage site consensus motifs between N-terminal leader peptide (LP) and central SU subunit [26,27,28]. Unlike processing of the SU- and TM subunits separation of LP- and SU subunits is not essential to virion infectivity but has some influence on the efficiency of particle release [26,27]. A processing of the viral glycoprotein by the viral protease, as reported for some orthoretroviruses, has not been detected during the transport of the FV Env precursor through the secretory pathway to the plasma membrane ([31,32] and unpublished observations).

During insertion in the secretory pathway and transport to the cell surface FV glycoproteins are heavily N-glycosylated [33]. For PFV a detailed mutagenesis analysis characterized posttranslational modification at 14 out of 15 potential N-glycosylation sites, some of which are evolutionary conserved amongst different FV species (Figure 3) [28,33]. The differential sensitivity to specific glycosidases indicated that all oligosaccharides on LP and TM are of the high-mannose or hybrid type, whereas most of those attached to SU, which contribute to about 50% of its molecular weight, are of the complex type [33]. Notably, individual inactivation of all PFV N-glycosylation sites revealed defects in intracellular processing, support of particle release, and infectivity only for three evolutionary conserved N-glycosylation sites [33]. Whether FV glycoproteins contain O-linked carbohydrates as reported for some orthoretroviral Env proteins is currently unknown [34,35].

For PFV the unusual biogenesis of the glycoprotein yields in particle-associated glycoprotein complexes consisting of a LP- (gp18^LP^) and TM subunit (gp48^TM^) with type II and type I membrane topologies, respectively, as well a luminal SU subunit (gp80^SU^), which probably is covalently connected by disulfide bridges to the TM subunit [15]. Very recently, it was reported that the PFV LP subunit is a substrate for cellular signal peptide peptidases, resulting in a processing in the hydrophobic membrane-spanning domain and indeed smaller LP cleavage products can be detected in lysates of FV infected cells and released particles [15,24,36]. The very recently reported additional processing of the FV glycoprotein leader peptide by signal peptide peptidase might reveal such an unexpected function. The situation is reminiscent to MMTV where the signal peptide targeting the glycoprotein to the secretory pathway is reported to function as a Rev-like RNA export factor, after being processed by signal peptidase and released into the cytoplasm [37,38]. To date such a factor has not been identified for FVs. It is therefore tempting to speculate that the FV Env LP cleavage product may possess similar functions in the viral RNA export as the signal peptide of the MMTV Rem and Env proteins.

### 3.2. Subcellular Localization of FV Budding

FV egress shows many features of orthoretroviruses following a B/D type assembly strategy (Figure 1). This includes assembly of capsids, consisting of FV Gag protein and viral genomic RNA (vgRNA), at a pericentriolar region, which was demonstrated to represent the centrosome or microtubule organizing center [39]. Similar to MPMV, FV Gag is targeted to the centrosome via a cytoplasmic targeting and retention signal (CTRS) found in the capsid protein (Figure 3) [6,40]. Transport of Gag or Gag-RNA complexes to the centrosome is dependent on microtubules of the host cell [39].

FV budding is reported to occur at different cellular membranes/locations [12,19,41]. All known FV species with one exception are thought to bud predominantly into intracellular compartments, a phenotype that correlates with an ER retrieval signal located at the C-terminus of the respective glycoproteins (Figure 3) [28,42,43]. Electron microscopy analysis of equine FV (EFV) infected cells suggests that it is the only FV species known to date that buds exclusively at the plasma membrane, which is explained by the lack of a C-terminal ER retrieval motif in EFV Env [28,41]. However, budding at the cell surface is also observed for other FV species, although at a much lower level as seen into apparently intracellular vacuolar structures [12,44]. However, the nature of these intracellular vacuolar budding compartment is poorly defined. Initial reports suggested them to represent ER-derived compartments, in line with a retrieval signal-mediated retention of the FV glycoprotein in the ER [12,44]. To date only for PFV the subcellular localization of the intracellular budding site was investigated in greater detail in infected Hela cells, by immunostaining of viral structural proteins and colocalization analysis with organelle specific markers [39]. The results suggest PFV budding to take place at membranes of the *trans*-Golgi network (TGN). PFV Gag and Env localized both with similar efficiency to TGN components and not to components of the pericentriolar region (unlike MPMV), the ER, late endosomes or multivesicular body (MVB). How FV particles budding into intracellular compartments transit to the cell surface is currently unclear, since no virion-containing vesicles fusing with the plasma membrane were observed in infected cells [12,39]. Only in cells expressing a specific PFV Env mutant, having the membrane-spanning-domain of the TM subunit replaced by MuLV and displaying a cell surface transport-deficient phenotype, vesicles containing single or few budded virions were observed [12]. Particle release into the supernatant of cells expressing this PFV Env mutant was not detectable, and budding structures were only observed at intracellular membranes but not at the cell surface. These virion-containing small vesicles might represent transport vesicles that are naturally employed after budding into intracellular compartments for rapid release, stuck in their transit to the cell surface.

Notably, FV infections are highly cytopathic *in vitro*, leading to a strong cytoplasmic vacuolization of most host cells and pronounced syncytia formation, as a consequence of FV glycoprotein-mediated cell-cell fusion [16,39] Therefore, it has to be clarified whether this may lead to changes in the intracellular architecture and an altered localization of endogenous cellular organelle markers, thereby complicating the characterization the FV budding sites in studies using infected cells. Furthermore, the notion of predominant intracellular budding of most FVs deduced from multiple electron microscopy studies of FV expressing cells should also be taken with caution due FVs cytopathogenicity inducing cell morphology alterations. The putative intracellular budding sites of most FVs may represent plasma membrane invaginations, similar to budding structures reported for HIV in infected macrophages [2,45]. Alternatively, FV particles found in intracellular vacuolar structures in electron micrographs may have been endocytosed after release at the membrane, as reported for HIV in certain cell types [46].

### 3.3. Details of FV Gag-Env Interaction

**Figure 4:**
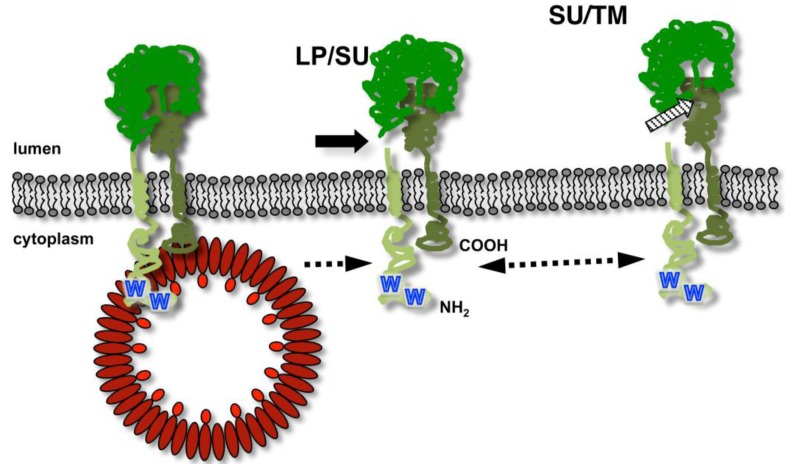
The specific foamy virus capsid – glycoprotein interaction essential for viral budding. Schematic illustration of FV precursor processing after insertion into the secretory pathway and interaction with the viral capsid (in red) essential for budding of virions. The order of LP/SU (solid arrow) and SU/TM (striped arrow) processing resulting in the different Env subunits (indicated in different shades of green) has not been defined. N-terminus (NH2) and C-terminus (COOH) are indicated. The essential WxxW motif in the LP CyD is indicated by blue W characters.

The unusual biosynthesis of the FV glycoproteins already indicates that they have additional functions in the replication strategy of FVs, besides determining the virus tropism and facilitating target cell entry by interacting with host cell receptor molecules. The Env LP subunit is a physical constituent of the particle-associated glycoprotein complex, which suggests that it might be involved in particle morphogenesis in addition to targeting the glycoprotein precursor to the secretory pathway [15,25]. Indeed, it was experimentally confirmed for PFV and FFV that the Env LP subunit harbors the main determinants responsible for the glycoprotein-dependent egress of FV virions. In particular, the N-terminus of the cytoplasmic domain of LP, containing an evolutionary conserved WxxW motif, is essential for a specific interaction with the capsid protein during the budding process (Figure 4) [15,25]. Biophysical and co-precipitation approaches using recombinant Gag and Env domains or peptides suggest a direct interaction between the Env LP subunit and the capsid protein, that appears to be sufficient for promoting particle release [24,25,47]. However, the N-terminal region of the hydrophobic membrane-spanning domain of the PFV Env TM subunit was also reported to influence the Gag-Env interaction [12]. However, it is currently unclear whether modifications in this part of Env abolish particle egress by direct inactivation of an interaction-domain with Gag. Instead, alternative membrane anchorage of the PFV Env may induce changes in the glycoprotein that indirectly prevent the LP domain-mediated physical interaction with Gag either by inducing global structural changes or altering intracellular trafficking or distribution of Env [12,48].

The determinants of FV Gag essential for the interaction with the cognate Env protein are only poorly defined [18,24,25,49,50,51,52]. Previous studies suggest that they reside in the N-terminal part of the Gag protein. It was demonstrated that the N-terminal 297 aa of PFV Gag, containing the essential L domain, are sufficient for an efficient Gag release into the supernatant when coexpressed with PFV Env [18,52]. Furthermore, small deletions within the N-terminal part of the protein abolished capsid egress and N-terminal fusion of protein tags to PFV Gag appear to interfere with viral egress in tag size dependent manner [18,50,51]. In addition, surface plasmon resonance studies using recombinant N-terminal protein fragments of FFV Gag (corresponding to PFV Gag aa1-180) and FFV Env peptides or recombinant protein fragments (corresponding to PFV Env aa1-28 or aa1-61) suggest a direct low affinity interaction between both proteins [24,25]. Finally, recent experiments from our lab employing recombinant protein fragments and co-precipitation assays confirm a direct interaction between PFV Gag and PFV Env and suggest an essential role of a putative N-terminal coiled-coil domain in PFV Gag for the WxxW-motif-dependent binding to PFV Env LP subunit (Figure 2) [47].

## 4. Cellular Factors Involved in FV Budding

In numerous cases, the budding of enveloped viruses from the cells is influenced by or requires cellular factors [1,53]. For pericentriolar assembly of capsids and budding of virions at different cellular membranes in an FV infected cell, FV structural proteins and preassembled capsids must traffic along different pathways within the host cell. Like other viruses, these steps in viral replication are aided by certain cellular components, but inhibited also by some others. However, unlike other retroviruses such as HIV-1, few cellular factors are presently known that promote FV egress from infected cells or act as restriction factors in these processes.

One cellular factor known to interfere with FV particle egress to date is tetherin [54,55]. Tetherin is an interferon 1-inducible transmembrane protein originally identified to inhibit HIV-1 release by tethering virions to the cell surface [56,57]. Later, it was demonstrated that tetherin has a broad antiviral activity inhibiting the release of various kinds of retroviruses, including FVs, and other viruses such as filoviruses [1].

Unlike inhibitory factors, several cellular factors or machineries promoting or being essential for FV release have been identified so far. For example, the cytoskeleton of the host cells is an important structure for viral protein trafficking and particle release, as observed for other retroviruses. Incubation of infected cells with microtubule-depolimerizing drugs such as nocodozale or colchicine strongly interferes with trafficking of FV Gag to and capsid assembly at the centrosome, as well as particle release [39,58,59]. PFV Gag is reported to interact with a light chain of dynein motor proteins through a conserved putative coiled-coil domain in N-terminal part of the protein during virus entry [58]. This suggests the use of these motor proteins for intracellular trafficking of the Gag protein and capsids. Notably, inactivation of this Gag motif also prevents centrosomal localization of de novo synthesized Gag upon transfection of cells. Whether the same motors are indeed utilized for assembly and/or egress as well has not been investigated in further detail. 

Similar to other retroviruses FVs were also found to hijack proteins of the vacuolar protein sorting (Vps) machinery for the budding and fission steps of virion egress [52,60,61,62]. Finally, the cellular ubiquitination machinery, also implicated in participating in cellular cargo protein sorting, interacts with FV virions during egress, resulting in posttranslational modification of FV structural proteins [63,64]. Our knowledge on the interaction of FVs with both types of cellular machineries is summarized in the next paragraphs.

### 4.1. The ESCRT Machinery

For all retroviruses, including foamy viruses, a set of proteins belonging to endosomal sorting complex required for transport (ESCRT) machinery is involved in particle egress. The ESCRT components are naturally utilized by the cell for sorting of cargo into multivesicular bodies (MVBs), a process that involves trafficking of proteins through various intracellular compartments [65]. Components of ESCRT take part in enabling cargo-enriched vesicles to bud into the lumen of MVBs and mediate the scission events that lead to final release of vesicles into MVB space, which is topologically reminiscent to virus budding. Also, this machinery is involved in final steps of cell division and specializes in mediating membrane scission events. As obligatory parasites, enveloped viruses have found a way to hijack the system by directing some of its components to enable budding of virions from the cells [2,66].

Retroviruses are able to recruit ESCRT components during budding via interactions mediated by their capsid precursor protein. Each retroviral Gag contains, at different locations throughout the protein, one or more of specific motifs named “late assembly” or L domains due to their central role in promoting late events in intracellular virus life cycle. Until present, three major L-domain sequences essential for position-independent ESCRT complex recruitment have been identified [67,68]. P(T/S)AP motifs initially characterized in HIV-1 Gag, but also present in other retroviral capsid precursors, enable budding on account of interaction with Tsg101, a component of the ESCRT I complex. (L)YPx_n_L motifs, the best characterized one found in equine infectious anemia virus (EIAV) and HIV-1 Gag proteins, utilize interactions with AIP-1/ALIX to recruit ESCRT III complex components for budding. PPxY motifs found in MuLV, HTLV-1 and avian sarcoma/leukosis virus (ASLV) capsid precursors or the HBV core protein promote budding by binding to the WW domains of a subset of NEDD4-like HECT ubiquitin ligases. However, the mechanism of ESCRT III component recruitment by these motifs remains elusive. Multiple, possibly redundant pathways for linking HECT ubiquitin ligases with the core ESCRT machinery are discussed. A fourth motif, FPIV (consensus ØPxV;_in which Ø is any aromatic amino acid) found in the M protein of paramyxovirus simian virus 5 was reported to have L domain activity and complement the budding defect of L-domain deficient HIV-1 Gag [69]. However, its cellular interaction partner is unknown and other viruses using a FPIV L domain motif have not been identified to date. Notably, many retroviral Gag proteins contain more than one type of L-domain motif and therefore can presumably use alternative pathways of intracellular trafficking involved in the budding process [1,70]. Furthermore, different requirements of particular retroviruses for individual ESCRT complexes are known, e.g., HIV utilizes ESCRT-I but not ESCRT-II while ASLV utilizes ESCRT-II but not ESCRT-I complexes. Markedly, regardless of the individual ESCRT component hijacked by the different retroviruses, all the budding strategies involve a common requirement. This is the dependence on ESCRT-III complex and catalytically-active ATPase Vps4, which enables final pinching off of a virus particle and recycling of ESCRT components recruited [1,68].

In case of FVs, motifs with perfect or some homology to the three major L domains were initially identified in the PFV Gag protein [52,62]. However, only the PSAP motif has been shown by mutagenesis analysis to act as a “true” late domain and to mediate efficient PFV budding via interaction with Tsg101. For the other two motifs, a YEIL and a PPPI motif, the suspected L domain function could not be confirmed. Later on a function as an interaction-domain influencing capsid morphology was reported for the YEIL motif, in context of a conserved (Y/F)xxLGL consensus motif present in all FV species [71]. The PPPI motif has no clear function attributed yet, although its mutation severely reduces viral infectivity without affecting particle release [52,62]. Another evidence supporting the notion that PFV particle budding is dependent on ESCRT pathway is the absolute requirement of the functional Vps4 protein. Overexpression of a dominant negative form of this ATPase in virus-producing cells led to a complete inhibition of release of particles with a functional PSAP motif [52,62].

Notably, all experimental data on the ESCRT recruitment by FVs has been obtained only for PFV. All primate FVs contain one to three P(S/T)AP L domain motifs (Figure 2B). Whereas these motifs are located in a central region of most primate FVs, spider monkey FV (SFVspm) Gag harbors an additional motif in the C-terminal p3^Gag^ subunit and the single PSAP motif of orangutan Gag is placed at the C-terminus of the p68^Gag^ subunit (Figure 2B). Interestingly, all non-primate FV species lack P(S/T)AP L domain motifs. Although unlikely, this raises the possibility that budding of these FVs is ESCRT-independent as reported for some other viruses [68]. Alternatively, and more likely, the capsid precursor proteins of non-primate FVs species may harbor yet uncharacterized L domain motifs. Notably, an additional PPRY motif that matches the PPxY L-domain consensus motif is found in the C-terminal part of squirrel monkey FV (SFVsqu) Gag. This motif fits a (Q/R)P(Q/A/S/P)R(Y/L)G consensus motif located at this position in all FV species (Figure 2B). Furthermore, all FFV Gag sequences contain FPIV and FPRV peptides that are perfect matches of the ØPxV L-domain consensus motif of the paramyxovirus simian virus 5 M protein (Figure 2B).

### 4.2. The Ubiquitination Machinery

The cellular ubiquitination machinery is implicated to be involved in the release of different viruses, in particular those hijacking the ESCRT machinery [72,73,74,75]. This notion derives from the observation that monoubiquitination of cargo serves as a signal for recognition and trafficking mediated by the ESCRT machinery [76]. Furthermore, free ubiquitin and ubiquitinated Gag proteins were detected in various kinds of retroviral particles in an L-domain dependent manner [72,73]. Finally, late budding defects induced by proteasomal inhibitor treatment or L-domain deletion can be alleviated by direct fusion of ubiquitin to the C-terminus of Gag proteins [60,77]. However, whether direct ubiquitination of retroviral capsids or of cellular proteins are critical for ubiquitin-dependent viral particle release remains unclear.

In the course of characterizing the budding pathways of different PFV particle structures ubiquitinated glycoprotein subunits were readily observed in lysates of released PFV particles (see below) [63]. The lack of Gag ubiquitination noted in these studies was not surprising since most FV Gag proteins have the unusual feature that they are completely or almost devoid of lysine residues, the acceptor sites of ubiquitination [60,63,78]. Strikingly, FFV Gag is special in this respect among FV capsid precursors since it contains the largest number of lysine residues and has five out of eight lysines clustered in the p3^Gag^ domain. Zhadina *et al*. [61] took advantage of this observation and the paucity of lysine residues in PFV Gag to determine whether retroviral capsid precursor protein ubiquitination is generally required for enveloped virion release. They used an artificial virus VLP system comprising an Env-independent budding PFV Gag protein with attached N-terminal heterologous membrane-targeting domain and conservatively mutated the single lysine residue in the PFV capsid precursor [61]. With a mutant variant of this PFV Gag protein, having the natural PSAP L-domain motif replaced by a heterologous PPxY motif, they could demonstrate that for the PPxY L-domain motif and ubiquitin ligase activity dependent particle release, ubiquitination of Gag was dispensable. Thus, ubiquitination of transacting factor rather than viral proteins appears critical for ubiquitin-dependent enveloped particle release. In a follow-up study, they reported a flexibility in the ways in which the ESCRT machinery can be recruited and how ubiquitin can be co-opted to enable this, for example attachment to Gag or involved cellular proteins [60]. These results indicate that our current view on cellular components involved in retrovirus budding should be expanded, as the routes of host factor hijacking by viruses are probably more intricate than already identified. 

Notably, viruses in general and also retroviruses in particular utilize the cellular ubiquitination machinery for replication also in another way, namely for neutralization of cellular antiviral factors [1,79,80]. By different mechanisms, they induce attachment of polyubiquitin chains to these factors thereby driving them into proteasomal degradation. Examples for this are the proteasomal degradation of tetherin mediated by HIV-1 Vpu or the HIV-1 Vif induced inactivation of APOBEC3 proteins, which is encapsidated in retroviral virions and edits the viral genome during reverse transcription. Both cellular factors are also known to interfere with FV replication [54,55,81,82,83]. However, a defense mechanism of FVs against tetherin restriction has not been identified [54,55]. Furthermore, ABOBEC3 proteins are neutralized by the FV accessory protein Bet through binding and sequestration rather than routing the cellular proteins to proteasomal degradation [84,85].

## 5. Alternative and Artificial Budding of FV Particle Structures

### 5.1. Subviral Particles

As previously mentioned, FV replication cycle is highly related to orthoretroviruses, but some aspects are more similar to hepadnaviruses. For instance, hepadnaviral infection is characterized by massive overproduction of subviral particles (SVP) [8,9]. These are released empty vesicles that contain only viral glycoproteins but no viral capsids or genomes and are non-infectious. Hepatitis envelope protein (HBsAg) alone is sufficient for mobilization of cellular lipids, self-assembly, intracellular transport and secretion [86]. Budding of subviral particle occurs from intracellular, post-ER pre-Golgi membranes [87,88]. SVP of HBV reach a 1,000–100,000-fold higher concentration in serum than infectious Dane particles, which represents an advantage of the virus against the host’s immune system [89,90]. SVP might neutralize antibodies produced from the immune system to stem virus proliferation or lead to immune tolerance that allow persistent infection [90,91].

In 2003, subviral particles were first described for PFV [92] and FV Env expression was shown to be sufficient to induce SVP release (Figure 1). In contrast to hepadnaviruses only small amount of SVP in comparison to infectious particles are released. However, only limited knowledge about regulation strategies for subviral particles formation is available. Studies with PFV Env defined structural requirements for SVP release involving two essential and two inhibitory regions in the PFV Env LP and TM subunits (Figure 3A) [52]. In addition, ubiquitination of the glycoprotein at the N-terminal cytoplasmic domain (CyD) of its LP subunit was shown to regulate the level of SVP formation [63,64] (Figure 3). For PFV about one third of the particle-associated LP subunits were ubiquitinated at one or more lysine residues, detectable as LP variants with higher molecular weight stained with ubiquitin or LP-specific antibodies in Western blots of PFV particle lysates [63]. Inactivation of the first three or of all five potential ubiquitin attachment sites in PFV LP massively increased the release of SVP [63]. By contrast ubiquitination at the first or second, but not the other lysine residues of the LP CyD were alone sufficient to suppress PFV SVP [64]. Strikingly, LP subunit ubiquitination was detectable for PFV and SFVmac, but not for FFV Env [15,25,63].

Not only the cellular ubiquitination machinery is involved in egress of PFV SVPs. Unlike HBV SVP release, late components of the VPS machinery are also important for PFV SVP budding [64]. 

What potential functions SVP particles might have in natural FV infections is currently not known. Due to the low level of PFV SVP release observed, it seems unlikely that they serve as decoys for the immune system like HBV SVPs. It is possible that the low level PFV SVP release is only a byproduct of the unique glycoprotein-dependent egress strategy of infectious FV particles. They might be a consequence of intrinsic structural features of the FV glycoproteins for particle morphogenesis that are absent from orthoretroviral glycoproteins.

### 5.2. Glycoprotein-Independent Capsid Membrane Targeting

Unlike their orthoretroviral relatives, FVs normally depend on Gag-Env interaction for final release of particles from infected cells, because FV Gag lacks an authentic membrane-targeting signal [12,13,14]. However, artificial ways allowing release of VLPs without envelope being present have been developed for FVs. These systems, describing FV particulate structure release, have all utilized the same valuable observation by Pellman *et al.*, that attaching a heterologous myristoylation signal from Rous Sarcoma virus Src protein to various cellular proteins can retarget them to plasma membrane [93]. Rous sarcoma virus membrane targeting and –binding domain (MTD) is responsible for directing Gag from cytoplasm to plasma membrane [94,95]. Hence, experimental systems using FV Gag proteins tagged with heterologous membrane-targeting signals have developed and demonstrated that FV budding from the cell can be induced in the absence of Env coexpression by providing these modified Gag proteins [40,49,51,61,96].

The ability of N-terminally myristoylated PFV Gag to form VLPs budding from the cell was first described by Eastman and Linial [40]. In this study, the PFV Gag CTRS was characterized by using proviral constructs having different point mutations in the Gag CTRS motif. Inactivation of the CTRS resulted in the abolishment of PFV budding and release and a nuclear accumulation of the mutant Gag proteins. By replacing the first ten N-terminal amino acids of these PFV Gag CTRS mutants with a heterologous MTD, the Src-myristoylation signal (v-src), the cellular distribution of Gag was altered leading to a plasma membrane localization of the Gag mutants. Furthermore, PFV budding was rescued at levels similar to or even higher than wild type and was no longer dependent on the coexpression of the cognate glycoprotein. However, these v-src-tagged viruses were non-infectious, independently of whether the FV glycoprotein was coexpressed or not.

Further investigation of the particle morphogenesis of PFV Gag variants tagged with different plasma membrane localization signals showed, however, that the modifications did not alter the location of intracellular PFV capsid assembly in presence of an intact CTRS [51]. The presence of a functional FV Gag CTRS appears to dominantly target capsid assembly to the pericentriolar site in the cell. The non-infectious phenotype of the MTD-tagged virions correlated with aberrant capsid morphologies, most likely as a consequence of reduced Pol incorporation and decreased Gag processing. 

Other studies using additional membrane localization signals, such as fyn or lck myristoylation and palmitoylation motifs or the complete matrix (MA) domains of HIV-1 or MPMV Gag, confirmed Env-independent particle release capabilities of the tagged Gag proteins of PFV and FFV [49,61,96]. As originally reported by Eastman *et al*., several of the alternative MTDs display a severe interference with FV virion infectivity, capsid morphogenesis and Pol incorporation [40,96]. Notably, only targeting of FV Gag to the plasma membrane and not to endosomal membranes is compatible with budding [96]. Another study with FFV Gag suggests that replacement of N-terminal FV Gag sequences by heterologous MTDs is less well tolerated than their N-terminal addition [49]. This is in line with a mutagenesis analysis of N-terminal MTD-tagged PFV Gag that demonstrated the interference of some MTD sequences with PFV morphogenesis when replacing the N-terminus of Gag [51]. However, a recent characterization of the Env interaction domain in PFV Gag indicates that the N-terminal 10 aa of PFV Gag are dispensable for Env-dependent morphogenesis and egress [47].

Taken together, these studies demonstrate that addition of heterologous plasma membrane targeting signals can compensate the lack of an endogenous MTD in FV Gag proteins and enable FV VLP release as inherent to orthoretrovirus Gag proteins. This also indicates that FV Gag harbors all other structural motifs necessary for capsid assembly and egress. Furthermore, the strong interference of permanent membrane association induced by MTD tagging of FV Gag proteins, due to their naturally limited proteolytic precursor processing features, strongly indicates that native FV virion morphogenesis includes only a transient membrane association, naturally mediated by the specific interaction with the cognate glycoprotein.

### 5.3. Pseudotyping

The term pseudotyping describes a phenomenon of viruses obtaining heterologous envelope proteins, a quite frequent occurrence upon infection of one cell with two different viruses. The primary consequence of such an event is broadening or narrowing of the natural virus host range, due to the features of the newly obtained envelope. 

Clearly, the ability to pseudotype different virus particles represent a valuable tool for development of viral vector systems, as the possibility of altering virus natural specificity opens vector applications to previously non-permissive cells and tissues [97,98]. Furthermore, pseudotyped viruses can have a safety advantage. For several retroviruses pseudotyping with heterologous Env proteins is reported [99]. For example, pseudotyping of lentiviral vectors with vesicular stomatitis virus glycoprotein (VSV-G) is the first used combination for pseudotyping lentiviruses. The resulted broad tropism and stability warrant the widely use of these vectors.

Due to the specific interaction of FV Gag and Env proteins, which is a prerequisite for particle egress, FVs are naturally resistant to pseudotyping by heterologous glycoprotein, even from other retroviruses [12,13,14]. Only very recently, a system for pseudotyping PFV particles has been described [100]. The approach that finally enabled PFV particles to be pseudotyped is based on using a system, where the apparently transient interaction of FV Gag and FV Env is substituted by an inducible, small-molecule controlled heterodimerization (HD)-system. This system enabled pseudotyping of PFV particles by VSV-G for instance. More precisely, PFV capsid protein was fused to one HD domain (HDD), while another HDD was fused to a heterologous MTD, such as the HIV-1 Gag MA subunit. Particles produced by coexpression of these components with the different envelope proteins, such as a naturally interaction-deficient PFV Env mutant or heterologous glycoproteins like VSV-G, displayed infectivity comparable to wild type PFV. Furthermore, PFV particles pseudotyped with heterologous glycoproteins were able to infect previously non-permissive PFV cell lines. Thus, successful pseudotyping of PFV capsids, opening new possibilities for FV vector design, has finally been achieved.

## 6. Conclusions

The last ten to fifteen years of FV research has shown enormous progress in our understanding of how this type of retroviruses replicate within their host cells. The viral egress strategy these viruses have chosen in particular highlights their unique position among retroviruses. It demonstrates that they are at the interface between retroviruses and hepadnaviruses. In this review we summarized the current knowledge on the FV budding and release processes. In our opinion, many of the unique features characterized for these processes that deviate from the general orthoretroviral pathways are based on the unusual biosynthesis and special features inherent to the FV structural proteins. The strict requirement of an intimate interaction of the capsid and glycoprotein for membrane association and budding, as a consequence of a missing membrane targeting signal in FV Gag and the unusual posttranslational processing of the Env protein, is probably the best example for this. Although it is known that both proteins influence each other’s intracellular distribution and trafficking, the mechanisms regulating it and their interaction prior to budding and release is still poorly characterized. Another enigma that remains unsolved is how FV particles, for most FV species apparently budding predominantly at intracellular membranes, exit the host cell. Are these budding sites indeed intracellular compartments, or not? Is FV budding the rate-limiting step in egress, and are subsequent post budding steps so efficient and fast that we have not observed them until now in ultrastructural studies? Analysis of FV egress with fluorescently-tagged structural proteins by live-cell imaging in real-time that recently became available also for FVs might shed more light on this in the future.

We are convinced that further examination of the biogenesis, trafficking and intracellular distribution of the FV Gag and Env but also the characterization of yet unknown cellular interaction partners will surface additional features and/or surprising functions of these proteins in the viral replication cycle. Enlarging our knowledge of the basic biology of these unusual retroviruses will also have an important impact in fostering their further development as gene transfer tools and potential applications in therapeutic strategies to cure various diseases.
